# Low-Temperature Thermomechanical Consolidation of Aluminosilicate Sorbents Impregnated with Model Oil-Containing Radioactive Waste

**DOI:** 10.3390/ma19183893

**Published:** 2026-09-12

**Authors:** Yerbolat Koyanbayev, Viktor Baklanov, Nuriya Mukhamedova, Arman Miniyazov, Igor Sokolov, Dilyara Belgibayeva, Ospan Oken, Aisara Sabyrtayeva, Anel Raiko

**Affiliations:** 1 National Nuclear Center of the Republic of Kazakhstan, Kurchatov 071100, Kazakhstansabyrtayeva@nnc.kz (A.S.);; 2 Research School of Physical and Chemical Sciences, Shakarim University, Semey 071412, Kazakhstan

**Keywords:** non-flowing state, oil-containing radioactive waste, aluminosilicate sorbents, thermomechanical consolidation, materials characterization

## Abstract

This paper investigates the possibility of pressure-assisted thermomechanical consolidation and thermal treatment without applied pressure during the low-temperature consolidation of oil-saturated aluminosilicate sorbents for their subsequent application in the conditioning of liquid oil-containing radioactive waste (RAW). The Premium and Absorbent sorbents saturated with transformer oil not containing radionuclides were used as model RAW systems. It was established that pressure-assisted thermomechanical consolidation leads to the formation of a denser structure and a reduction in hydrocarbon-phase losses compared with thermal treatment without applied pressure. For Premium, mass losses decreased from 0.74 to 0.35 g (by 53%), and for Absorbent, from 0.75 to 0.47 g (by 37%), and the consolidated samples were characterized by a more homogeneous microstructure and increased resistance to exudation. It was established that the efficiency of hydrocarbon-phase retention is determined by both the treatment conditions and the mineral composition of the sorbent. The obtained results indicate the prospects of low-temperature pressure-assisted thermomechanical consolidation at a temperature of 150 °C, a pressure of 1.5 MPa, and a holding time of 20 s for the conditioning of oil-containing RAW without the use of additional binding components.

## 1. Introduction

Oil-containing liquid RAW generated during the operation and decommissioning of nuclear facilities [1,2,3,4,5,6,7] represents one of the most challenging categories of RAW. Its specificity is determined by the combination of radiation hazard and the physicochemical properties of the hydrocarbon phase, including fluidity, fire hazard, and its effect on radionuclide migration [8,9,10,11]. An additional problem is the radiation and thermal decomposition of organic components with the formation of gaseous products that may affect the long-term safety of storage facilities [12,13,14,15]. Therefore, the development of effective technologies for the conditioning of oil-containing RAW remains an important task. The relevance of this problem also increases during the decommissioning of nuclear facilities [16], such as the BN-350 reactor [17], which is accompanied by the accumulation of significant volumes of liquid RAW [18].

As is known, the flash point of transformer oils is approximately 135–150 °C [19,20,21,22], which significantly limits the application of high-temperature consolidation technologies and necessitates the development of low-temperature technologies for converting oil-containing RAW into a non-flowing state without preliminary removal of the hydrocarbon phase [9,15,23,24,25]. During pressure-assisted thermomechanical consolidation, uniaxial loading and temperature are applied simultaneously, which ensures rapid closure of interparticle pores and limits the release of volatile components [26,27,28]. During thermal treatment without applied pressure, preliminary pressing at room temperature is performed separately prior to thermal treatment, while subsequent heating is carried out without mechanical loading under atmospheric conditions [29].

Aluminosilicate sorbents based on bentonite, owing to their developed porous and layered structure, are capable of effectively retaining the hydrocarbon phase both on the particle surfaces and in the interlayer space of clay minerals [30,31,32,33], making them promising matrices for the conditioning of oil-containing RAW. It has previously been shown that pressure-assisted thermomechanical consolidation enables the production of dense aluminosilicate matrices with a low leaching rate during radionuclide immobilization [34,35]. Despite the availability of studies on spark plasma sintering for the production of ceramic matrices for radionuclide immobilization, the application of low-temperature thermomechanical consolidation to oil-saturated aluminosilicate sorbents without preliminary removal of the organic phase has not been described to date. Compared with cementation and geopolymerization, the application of which to oil-containing waste is limited by the chemical incompatibility of the organic phase with the binder and the decrease in strength with increasing organic-phase content [23,24,34], as well as with thermoplastic and glass–ceramic matrices, which require high-temperature treatment incompatible with the thermal sensitivity of the hydrocarbon phase. The proposed approach is based on the direct consolidation of the oil-saturated sorbent without prior extraction of the organic phase and without the addition of any additional binding components.

The aim of the present work is to investigate the consolidation process of aluminosilicate sorbents impregnated with a simulant of oil-containing liquid RAW by means of thermal treatment with or without applied pressure, as well as to determine the role of the mineral composition of the sorbent in the resistance to exudation and the structural integrity of the consolidated samples.

## 2. Materials and Methods

### 2.1. Materials

The commercial aluminosilicate sorbents Premium and Absorbent (USA) were used as mineral matrices. The materials consist of bentonite 90–100 wt.% with a crystalline SiO_2_ phase content of up to 10 wt.% (Table 1).

Sorbent Premium underwent additional industrial processing, including moisture removal using an air stream, which resulted in reduced dust formation and increased sorption capacity of the material. Sorbent Absorbent did not undergo such processing, which is consistent with its lower standard absorption capacity.

To develop the procedure for the thermomechanical consolidation of oil-containing RAW and to exclude radiation exposure to personnel, commercial transformer oil (GOST 982-80) without the addition of radionuclides was used as the model hydrocarbon phase. Transformer oil was selected as the model hydrocarbon phase because it is widely used in nuclear facility equipment and is one of the main sources of oil-containing RAW generated during its operation and decommissioning [8,9]. The characteristics of the oil used correspond to the typical composition of transformer oils used in nuclear power plant equipment [19,20,21,22] and are presented in Table 2.

For the purposes of the present study, the most significant characteristics of the oil are the flash point 135–150 °C, which determines the upper limit of the permissible consolidation temperature, and the kinematic viscosity 28–30 mm^2^/s at 20 °C, which ensures uniform impregnation of the porous structure of the sorbent due to capillary forces. The predominance of cycloparaffin hydrocarbons ~70% reflects the typical composition of transformer oils used in nuclear power plant equipment.

### 2.2. Sample Preparation

The sample preparation process included impregnation of the sorbents, grinding, and subsequent consolidation.

The aluminosilicate sorbents were impregnated with transformer oil at a temperature of (20 ± 2) °C. To ensure uniform contact of the oil with the entire volume of the sorbent, the mixture was periodically stirred. The impregnated granules were ground in a planetary ball mill (PM100, Retsch GmbH, Haan, Germany) at a ball-to-material mass ratio of 10:1 and a rotation speed of 200 rpm for 60 min. After grinding, 2 g of powder of each sorbent, Premium and Absorbent, was taken for each thermal treatment condition. The powders obtained after grinding were pre-pressed at room temperature (20 ± 2 °C) in a hydraulic press with a holding time of 10 min to obtain pressed samples prior to thermal treatment. The pressing pressure was determined empirically and was 4 MPa for Absorbent and 2 MPa for Premium. At a pressure exceeding 2 MPa, visible release of the liquid phase from the Premium sorbent was observed, whereas the Absorbent sorbent withstood pressures of up to 4 MPa without oil release. This difference is associated with the higher oil content relative to the mass of the solid matrix in the Premium sorbent. At the same oil content of 10 g, the sorbent mass was 20 g for Premium and 50 g for Absorbent, corresponding to sorbent-to-oil ratios of 2:1 and 5:1. After pre-pressing, the pressed samples were subjected to thermal treatment. Pressure-assisted thermomechanical consolidation was performed using a spark plasma consolidation unit, whereas thermal treatment without applied pressure was carried out using a muffle furnace. In both cases, initial pre-pressing at room temperature was a common step that formed the pressed pellet. During thermal treatment without applied pressure, no pressure was reapplied after pre-pressing; that is, heating was performed without load. During pressure-assisted thermomechanical consolidation, the pressed pellet was subjected to pressure again, this time simultaneously with heating and under vacuum:

1. Thermal treatment without applied pressure was performed in a LE 6/11/R7 furnace (Nabertherm, Lilienthal, Germany) at 150 °C for 60 min. The process was carried out in air at atmospheric pressure without the application of an external load. Temperature control was provided by an integrated microprocessor controller with a temperature maintenance accuracy of ±1 °C.
2. Pressure-assisted thermomechanical consolidation was performed on the “ISKRA” setup at 150 °C. The samples were held under vacuum at a residual pressure of 1 Pa and a constant uniaxial pressure of 1.5 MPa for 20 s.

The process parameters on the “ISKRA” setup were monitored using a thermocouple positioned in a technological hole in the graphite die. The temperature control accuracy of the control system was ±0.1 °C, and the pressure stability was ±0.1 MPa. Heating was carried out using a pulsed DC power supply with a current of 3000 A, a voltage of 5.5 V, and a pulse frequency of 30 kHz.

### 2.3. Analytical Methods

X-ray diffraction (XRD) analysis of the initial ground sorbents was performed using a D6 PHASER diffractometer (Bruker AXS, Karlsruhe, Germany) with CuKα radiation λ = 1.5406 Å. Measurements were performed over the 2θ range of 10–100° with a step size of 0.015°. Phase identification was performed using the PDF-4/Axiom 2025 database.

Morphological studies of the impregnated sorbents were performed by scanning electron microscopy (SEM) using a TM4000 Plus microscope (Hitachi, Tokyo, Japan) to evaluate the microstructure, degree of consolidation, surface morphology and visible structural defects of the samples after pressure-assisted thermomechanical consolidation and thermal treatment without applied pressure. Imaging was performed in backscattered electron (BSE) mode at an accelerating voltage of 15 kV and a pressure of 30 Pa without conductive coating. Due to the presence of the hydrocarbon phase retained in the sample pores after consolidation, charging artifacts and image drift occurred at magnifications above ×500; therefore, images were acquired at magnifications not exceeding ×500. SEM was used for comparative evaluation of the overall surface morphology, visible voids, degree of densification, and large cracks in the samples, while quantitative assessment of porosity was performed based on density data. The elemental composition was determined by energy-dispersive X-ray spectroscopy (EDS) using a Quantax 75 spectrometer (Bruker AXS, Karlsruhe, Germany) with a spectrum acquisition time of 15 min. EDS data processing was performed using ESPRIT 2.0 with automatic correction of matrix effects based on the Φ(ρZ) model and standardless quantitative analysis. The obtained results were used to evaluate the sample surfaces and the distribution of elemental composition after consolidation. In addition, EDS analysis was performed on the initial non-impregnated ground sorbents for qualitative comparison of the elemental composition before and after thermomechanical treatment.

Gravimetric analysis was performed using HT analytical balances with an accuracy of ±0.0001 g. The sample mass was measured after impregnation, pressing, and consolidation to quantitatively determine the hydrocarbon-phase losses at each stage and to calculate the total losses relative to the initial 2 g sample. The obtained data were used for comparative evaluation of the hydrocarbon-phase retention efficiency under the two thermal treatment conditions. Mass measurements at each processing stage were performed for a single sample for each investigated condition (n = 1). Repeated measurements to assess reproducibility were not performed in the present study.

To determine the exudation threshold of the consolidated samples, an exudation test procedure based on the principles of ISO 10414-1 (API 13B) [36] and the work of Holder et al. [37] was used. The test was conducted for comparative evaluation of the ability of the consolidated samples to retain the hydrocarbon phase under external mechanical loading. The procedure was adapted to account for the characteristics of the formed solid samples and was not considered a standardized test of the mechanical properties of the matrix. Unlike tests of fluids in filtration cells, the present study employed uniaxial compression of the prepared consolidated samples placed on filter paper using a hydraulic press, with a holding time under load of 60 min at room temperature. The procedure was used as a model assessment of the resistance of the consolidated material to the release of the free hydrocarbon phase under mechanical loading simulating loads arising during the storage, transportation, and subsequent handling of conditioned waste. The tests were performed at loads of 0.1 and 0.3 MPa, selected as model values simulating mechanical loading during the storage and transportation of conditioned waste. Any visually detectable oil release onto the filter paper or loss of structural integrity of the sample was considered a negative result, since the purpose of conditioning is to ensure a non-flowing state of the material that prevents the release of the hydrocarbon phase under mechanical loading. The mass loss of the samples after loading was additionally determined gravimetrically using the same procedure described above. The measured mass loss reflects the total change in the sample mass before and after loading and is not an isolated measurement of the mass of oil transferred to the filter paper.

The mechanical stability of the samples was evaluated by measuring Vickers microhardness in accordance with the international standard ISO 14705:2016 [38] using an automated Qness 60 M EVO system with a Qpix Control2 M optical analysis system (QATM, Golling an der Salzach, Austria). A diamond pyramid with an angle of 136° between opposite faces was used as the indenter. A load of 2.942 N was used for Premium and 9.807 N for Absorbent. These conditions provided clear indentations suitable for measuring the diagonals without compromising the surface integrity, with a holding time of 13–15 s. Ten indentations were made on each sample, with a distance between them of at least three diagonals. The mean microhardness values (HV ± SD) were calculated automatically using the Qpix Control2 M software. The tests were performed only for samples after pressure-assisted thermomechanical consolidation, since the samples after thermal treatment without applied pressure were brittle and fractured upon contact with the indenter.

The density of the consolidated samples was determined from their mass and geometric dimensions. The apparent density was calculated as the ratio of the sample mass to its volume. The degree of densification was determined as the ratio of the apparent densityVersion 2.5.1.0 of the consolidated sample to the density of the initial unimpregnated sorbent granules. The density of the initial (unimpregnated) sorbents was determined by hydrostatic weighing of five granules of each sorbent and was 1.61 ± 0.33 g/cm^3^ for Absorbent and 1.26 ± 0.25 g/cm^3^ for Premium.

## 3. Results and Discussion

Since the method for the consolidation of oil-saturated aluminosilicate sorbents using pressure-assisted thermomechanical consolidation has not been previously described, the working sorbent/oil ratio required separate justification. The selection of ratios of 2:1, 3:1, 4:1, and 5:1 was performed sequentially for each sorbent, starting with the lowest oil content, until two criteria were simultaneously achieved: visually complete impregnation of the granules without signs of unimpregnated material and the absence of free oil phase release under manual mechanical action on the impregnated material. The second criterion is of fundamental importance, since insufficient mechanical stability of the impregnated material may result in a situation where the sorbent visually absorbs the oil completely but does not retain it under subsequent mechanical action. This test is fundamentally different from the exudation test (Section 3.5), which was subsequently performed on the consolidated samples using a hydraulic press and fixed loads of 0.1 and 0.3 MPa.

For Premium, this criterion was achieved at a ratio of 2:1, while for Absorbent, it was achieved at a ratio of 5:1. The holding time was determined experimentally until complete absorption of the oil phase was achieved. For Premium, stabilization of the mass and the absence of free liquid on the surface were recorded on the 21st day. For Absorbent, this process proceeded more slowly, and a similar state was achieved by the 30th day. The difference in saturation kinetics is attributed to differences in the mineral composition and sorption capacity of the materials (Table 1). Sorbent saturation with oil proceeds through a capillary impregnation mechanism, the rate of which is determined by the viscosity of the hydrocarbon phase (28–30 mm^2^/s at 20 °C, Table 2) and the pore-space structure of the sorbent. The lower bulk density and larger specific volume of Absorbent (Table 1) indicate a more developed and, probably, less homogeneous pore structure, which slows the attainment of equilibrium saturation compared with Premium and explains the longer period required for mass stabilization.

### 3.1. X-Ray Diffraction Analysis

XRD analysis of the initial Premium and Absorbent sorbents was performed to determine their mineralogical phase composition and identify phase differences that may influence the retention of the hydrocarbon phase and the mechanical stability of the consolidated samples.

**Figure 1:**
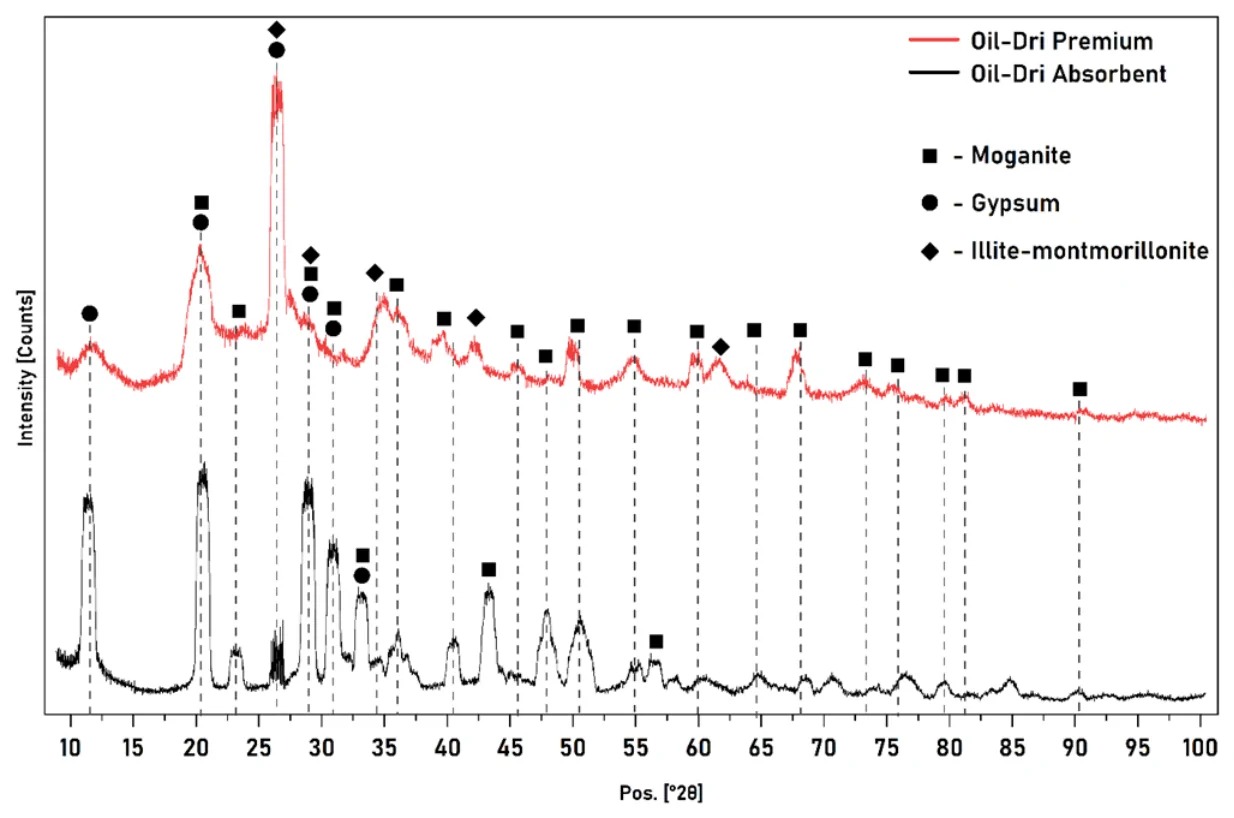
X-ray diffraction patterns of the initial ground unimpregnated sorbents Absorbent and Premium.

According to the X-ray diffraction analysis results, both sorbents are characterized by the same qualitative phase composition, including illite–montmorillonite (KAl_4_(Si, Al)_8_O_10_(OH)_4_·4H_2_O), gypsum (CaSO_4_·2H_2_O), and moganite (SiO_2_) (Figure 1).

For Premium, the most intense peaks correspond to illite–montmorillonite, indicating the predominance of the layered aluminosilicate clay material contribution [39,40]. In Absorbent, in contrast, an increase in the relative intensity of gypsum reflections is observed [41,42], which corresponds to its predominance in the phase composition.

The differences in the relative intensity of the reflections at the same qualitative composition are primarily due to different quantitative phase ratios, as well as the possible effect of additional thermal activation of Premium (moisture removal using an air stream), which may alter the degree of crystallinity and texture of the material without changing its qualitative phase composition. The additional broadening of the reflections is associated with grinding of the samples prior to measurement [43]. It should be noted that gypsum CaSO_4_·2H_2_O is not mentioned in the manufacturer’s specifications (Table 1), which indicate only bentonite at 90–100% and SiO_2_ at 0–10%. The presence of gypsum was identified by XRD as an additional result of the present study. For Premium, the XRD data generally correspond to the specified composition, since the identified illite–montmorillonite and moganite are consistent with the stated contents of bentonite and SiO_2_. For Absorbent, in contrast, the experimentally established predominance of gypsum is not reflected in the manufacturer’s specifications. The discrepancy between the composition stated by the manufacturer and the experimentally determined phase composition of Absorbent may be associated with variability in the mineral composition of the raw material between batches, as well as with the fact that the manufacturer’s specification data reflect the generalized chemical composition of the material. The possible influence of storage conditions or material aging on the phase composition was not investigated in the present study and cannot be excluded. The influence of the gypsum phase on the mechanical stability and fracture behavior of the consolidated samples is considered in Section 3.2 and Section 3.4. X-ray diffraction analysis was performed on the initial unimpregnated ground sorbents before impregnation and consolidation. The phase composition of the consolidated samples was not separately investigated, since the residual hydrocarbon phase forms an amorphous scattering background that masks weak reflections. It should be noted that dehydration of the water of crystallization of gypsum (CaSO_4_·2H_2_O), with its transformation into the hemihydrate, occurs in the range of 100–150 °C [44], which does not exclude partial transformation of the gypsum phase during thermal treatment without pressure (60 min at 150 °C). For illite–montmorillonite, mainly reversible loss of interlayer water is expected in this range, whereas dehydroxylation of structural OH groups begins at approximately 400 °C [45], which is substantially higher than the consolidation temperature. Direct confirmation of the phase composition of the consolidated samples by independent methods was not performed and is considered a task for future work.

### 3.2. Appearance and Morphology of the Samples

**Figure 2:**
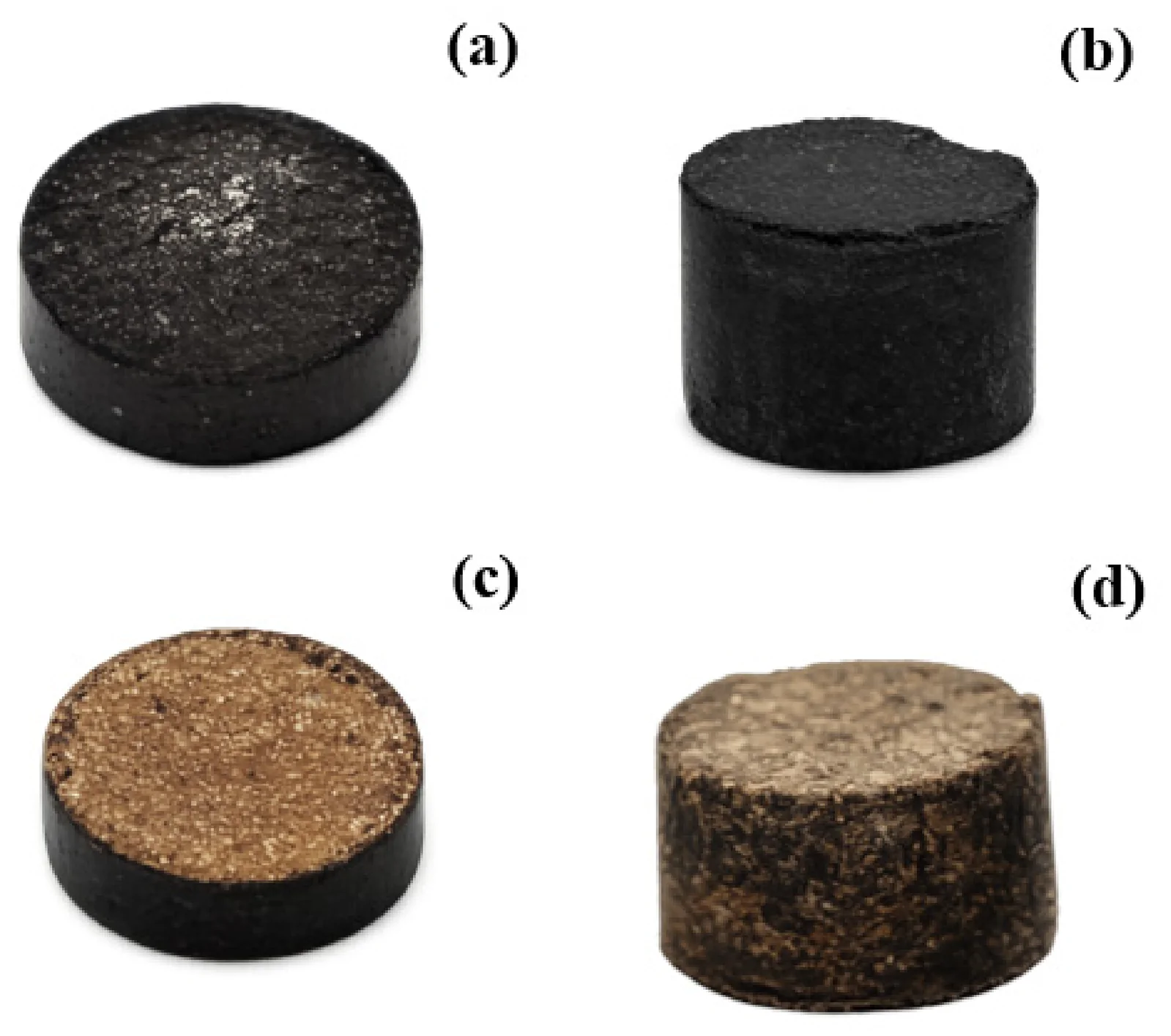
Appearance of the consolidated samples: (**a**) Premium, ISKRA setup; (**b**) Premium, muffle furnace; (**c**) Absorbent, ISKRA setup; (**d**) Absorbent, muffle furnace.

The samples after pressure-assisted thermomechanical consolidation had a lower height and larger diameter compared with the samples after thermal treatment without applied pressure (Figure 2). For Premium, after thermal treatment in the muffle furnace, the diameter and height of the sample were 1.18 and 0.815 cm, respectively, whereas after treatment using the ISKRA setup, they were 1.53 and 0.544 cm. For Absorbent, after thermal treatment in the muffle furnace, the diameter and height were 1.168 and 0.684 cm, respectively, whereas after treatment using the ISKRA setup, they were 1.509 and 0.408 cm. For both sorbents, pressure-assisted thermomechanical consolidation resulted in an increase in sample diameter and a decrease in sample height.

Pressure-assisted thermomechanical consolidation combines short-term thermal treatment with the simultaneous application of uniaxial pressure, which promotes effective densification of the structure and an increase in the degree of densification of the material compared with thermal treatment without applied pressure.

**Figure 3:**
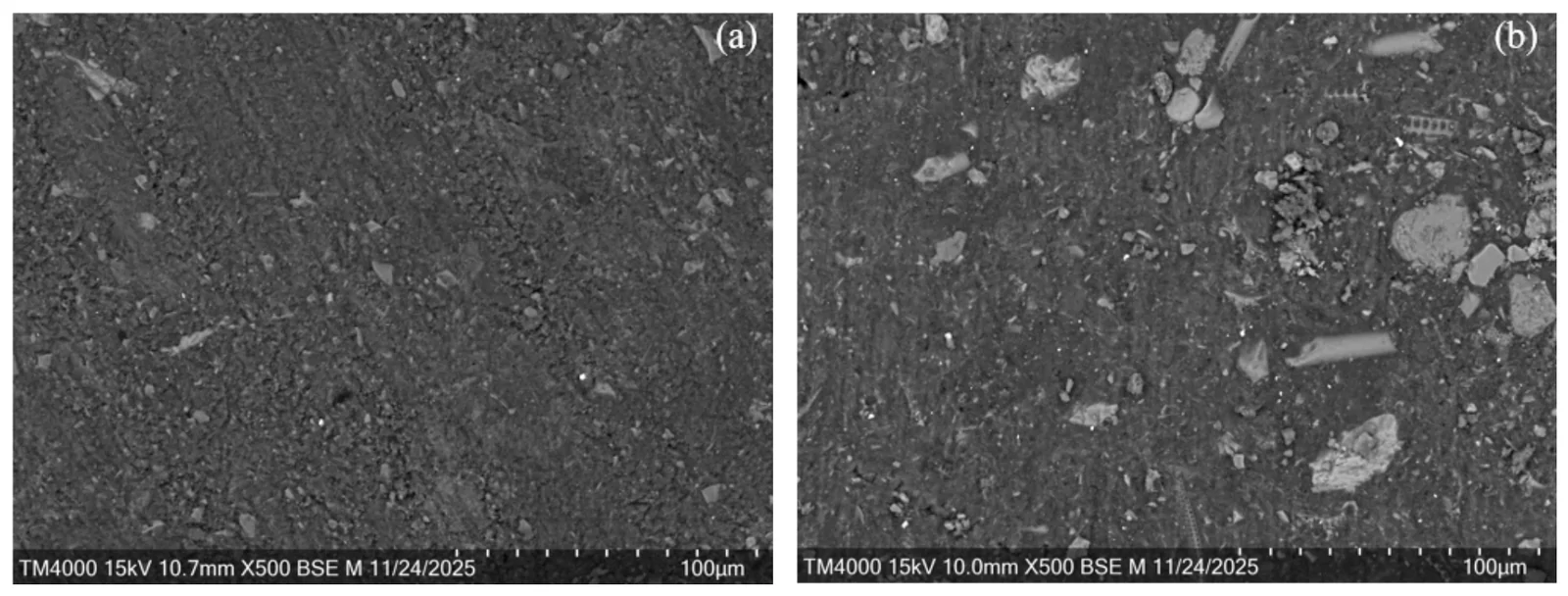

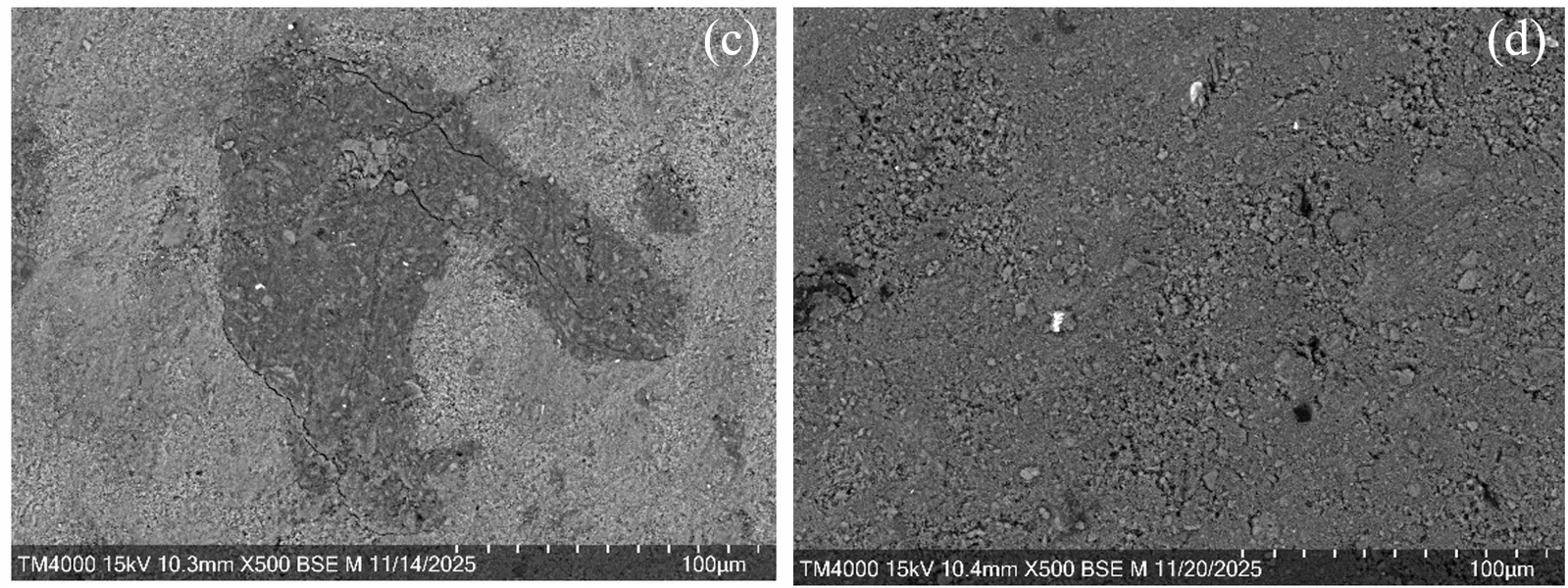
SEM images of the samples obtained at 150 °C: (**a**) Premium, ISKRA setup; (**b**) Premium, muffle furnace; (**c**) Absorbent, ISKRA setup; (**d**) Absorbent, muffle furnace.

As shown by the SEM images (Figure 3), the structure of the samples after pressure-assisted thermomechanical consolidation is characterized by more pronounced contact between the particles and a smaller number of visible voids compared with the samples after thermal treatment without applied pressure.

Premium is characterized by a homogeneous morphology. After pressure-assisted thermomechanical consolidation (Figure 3a), the surface is characterized by a smaller number of visible voids and more pronounced contact between particles compared with the sample after thermal treatment without applied pressure. This is consistent with the calculated density and porosity values obtained independently of SEM. In the sample after thermal treatment without applied pressure (Figure 3b), pronounced surface heterogeneity is observed, while the BSE images show bright contrasting inclusions of phases with a higher atomic number. The structure of Absorbent after pressure-assisted thermomechanical consolidation (Figure 3c) contains macrocracks, indicating brittle fracture of the surface during thermomechanical treatment. The tendency of Absorbent to brittle fracture is attributed to its phase composition. According to the XRD data, gypsum predominates in this sorbent, which is a brittle material with limited ability to undergo plastic deformation under pressure [46,47], and the EDS results are characterized by an increased content of Ca and S, which is consistent with the presence of the gypsum phase. The limited plasticity of the gypsum phase does not allow the material to compensate for mechanical stresses arising during consolidation through deformation, which leads to the formation of the macrocracks observed in Figure 3c. At the same time, local stress gradients arising from the application of pressure, redistribution of the residual hydrocarbon phase, and differences in the thermal behavior of the mineral components could also have influenced crack formation. Premium contains illite–montmorillonite, in the interlayer space of which diffusion and retention of hydrocarbon-phase molecules may occur [32], providing more effective retention of the hydrocarbon phase under thermomechanical treatment. The most porous morphology is observed for the Absorbent sample after thermal treatment without applied pressure (Figure 3d), which is attributed to the release of volatile components of the hydrocarbon phase.

### 3.3. EDS Analysis of Elemental Composition

To evaluate changes in the elemental composition of the sorbents during treatment, EDS analysis was performed on the initial unimpregnated sorbents and on the consolidated samples (Table 3).

**Table 3:** EDS analysis results for the initial (unimpregnated) sorbents.

Element	Premium Mass %/at. %	Absorbent Mass %/at. %
C	17.14/24.49	9.75/14.95
O	53.65/57.54	59.95/69.01
Mg	2.38/1.68	1.11/0.84
Al	5.70/3.63	0.93/0.63
Si	19.69/12.03	1.70/1.11
S	0.04/0.02	10.84/6.22
Ca	0.78/0.33	15.59/7.16
K	0.63/0.28	0.14/0.06

The increased carbon content in individual analysis areas may be associated with the presence of a residual hydrocarbon phase; however, quantitative determination of the oil content based on EDS data was not performed due to the possible contribution of the carbon substrate. The EDS data for the initial sorbents are consistent with the XRD results. The Ca-to-S ratio in Absorbent is close to the stoichiometric ratio of gypsum, CaSO_4_·2H_2_O, which is consistent with its predominance in the phase composition. For Premium, the low contents of Ca and S combined with elevated Si and Al contents are consistent with the predominance of illite–montmorillonite identified by XRD.

The EDS analysis results (Table 4) reflect differences in the elemental composition of the samples depending on the sorbent type and thermal treatment condition.

**Table 4:** EDS analysis results of the elemental composition of the samples, wt.% and at.%.

Element	Premium (ISKRA) Mass %/at. %	Premium (Muffle Furnace) Mass %/at. %	Absorbent (ISKRA) Mass %/at. %	Absorbent (Muffle Furnace) Mass %/at. %
C	26.80/37.99	38.27/50.85	14.27/23.00	19.30/30.47
O	39.91/42.47	34.05/33.96	42.88/51.88	40.08/47.50
Mg	2.22/1.55	2.20/1.44	3.21/2.56	1.33/0.88
Al	5.30/3.35	4.09/2.42	2.68/1.92	0.98/0.69
Si	21.90/13.27	17.81/10.12	7.74/5.34	3.10/2.09
S	-	-	11.47/6.93	14.19/8.39
Ca	0.84/0.36	0.82/0.33	15.48/7.47	20.64/9.77
Fe	2.39/0.73	2.04/0.58	1.47/0.51	0.57/0.19
K	0.64/0.28	0.72/0.29	0.80/0.40	-

According to the EDS analysis data (Table 4), the Premium (MP) sample contains iron at 2.04 wt.% and a small amount of calcium at 0.82 wt.%. Considering the higher atomic number of iron, its presence is associated with the appearance of bright contrast in the BSE images. EDS mapping of the selected surface area of Absorbent with structurl defects shows a distribution of Ca and S consistent with the presence of the gypsum phase (Table 4).

The Premium samples are characterized by increased Si and Al contents, which is consistent with the XRD data indicating the presence of illite–montmorillonite. The Absorbent sample after thermal treatment without applied pressure is characterized by a high combined content of Ca and S, corresponding to the presence of gypsum identified by XRD. The relatively low Ca content in the Absorbent sample after pressure-assisted thermomechanical consolidation may be associated with the heterogeneous distribution of the gypsum phase in the selected analysis area. The combined results of SEM, EDS, and XRD indicate that the use of Premium in combination with pressure-assisted thermomechanical consolidation promotes the formation of a denser and more homogeneous structure. The higher carbon content in the Premium samples compared with Absorbent indirectly reflects more effective retention of the hydrocarbon phase. The interparticle pores are able to close under pressure before the hydrocarbon phase migrates to the surface [26,46], while the interlayer space of illite–montmorillonite additionally contributes to the retention of hydrocarbon molecules [30].

### 3.4. Density and Degree of Densification

The initial mass of all sorbent portions before pressing was 2.00 g. Gravimetric monitoring of the samples at different stages of treatment revealed a decrease in the mass of all investigated compositions (Table 5), which was attributed to the removal of volatile components of the hydrocarbon phase [24]. Mass losses were recorded separately at the pressing stage and at the thermal treatment stage, and the total loss relative to the initial mass was also calculated. In the following discussion, the term “mass loss” refers to the total loss from the initial mass to the mass after thermal treatment, unless otherwise specified. The variation in mass losses during pressing is associated with the non-uniform distribution of oil in an individual 2.00 g powder portion.

**Figure 4:**
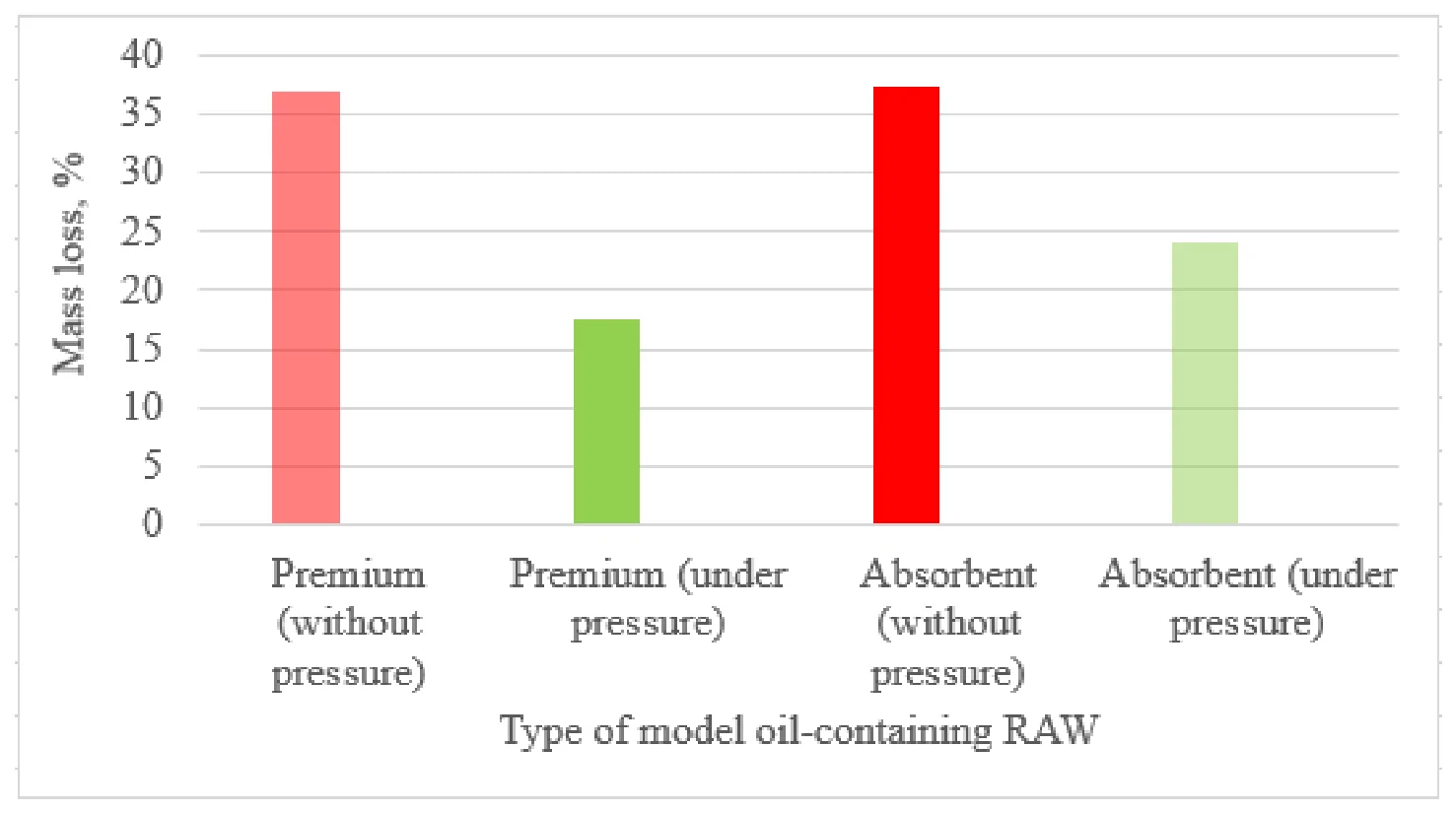
Total mass losses of Premium and Absorbent samples after treatment using the ISKRA setup and muffle furnace.

The apparent density was calculated as the ratio of the sample mass to its volume. The degree of densification was determined relative to the density of the initial unimpregnated sorbent granules. Differences in hydrocarbon-phase losses reflect the effect of the treatment conditions on oil retention within the sorbent structure (Figure 4).

Samples after pressure-assisted thermomechanical consolidation are characterized by higher density and a higher degree of densification compared with samples after thermal treatment without applied pressure. The highest apparent density was observed for the Absorbent sample after pressure-assisted thermomechanical consolidation, 2.099 g/cm^3^, which is associated with the higher true density of the initial sorbent, 1.606 g/cm^3^, compared with 1.255 g/cm^3^ for Premium, rather than with more effective structural densification. At the same time, the calculated degree of densification was comparable for both sorbents. According to the SEM data, the Absorbent sample after pressure-assisted thermomechanical consolidation contains macrocracks, whereas Premium retains a more homogeneous structure, which contributes to more stable retention of the hydrocarbon phase.

Differences in hydrocarbon-phase losses are explained by a combination of factors. Illite–montmorillonite in Premium retains hydrocarbon molecules in the interlayer space, with rapid diffusion prevailing at the initial stage, followed by slower viscous flow [30]. The short duration of the simultaneous treatment limits the second stage and promotes better oil retention. Uniaxial pressure additionally rapidly closes the interparticle pores and limits the release of the hydrocarbon phase [26,46], whereas the more pronounced presence of the gypsum phase in Absorbent may contribute to the formation of macrocracks during pressure-assisted consolidation. Despite the vacuum in the chamber at 1 Pa, which creates conditions for oil evaporation, the total mass losses after pressure-assisted thermomechanical consolidation were lower than after thermal treatment without applied pressure.

### 3.5. Exudation Tests

**Figure 5:**
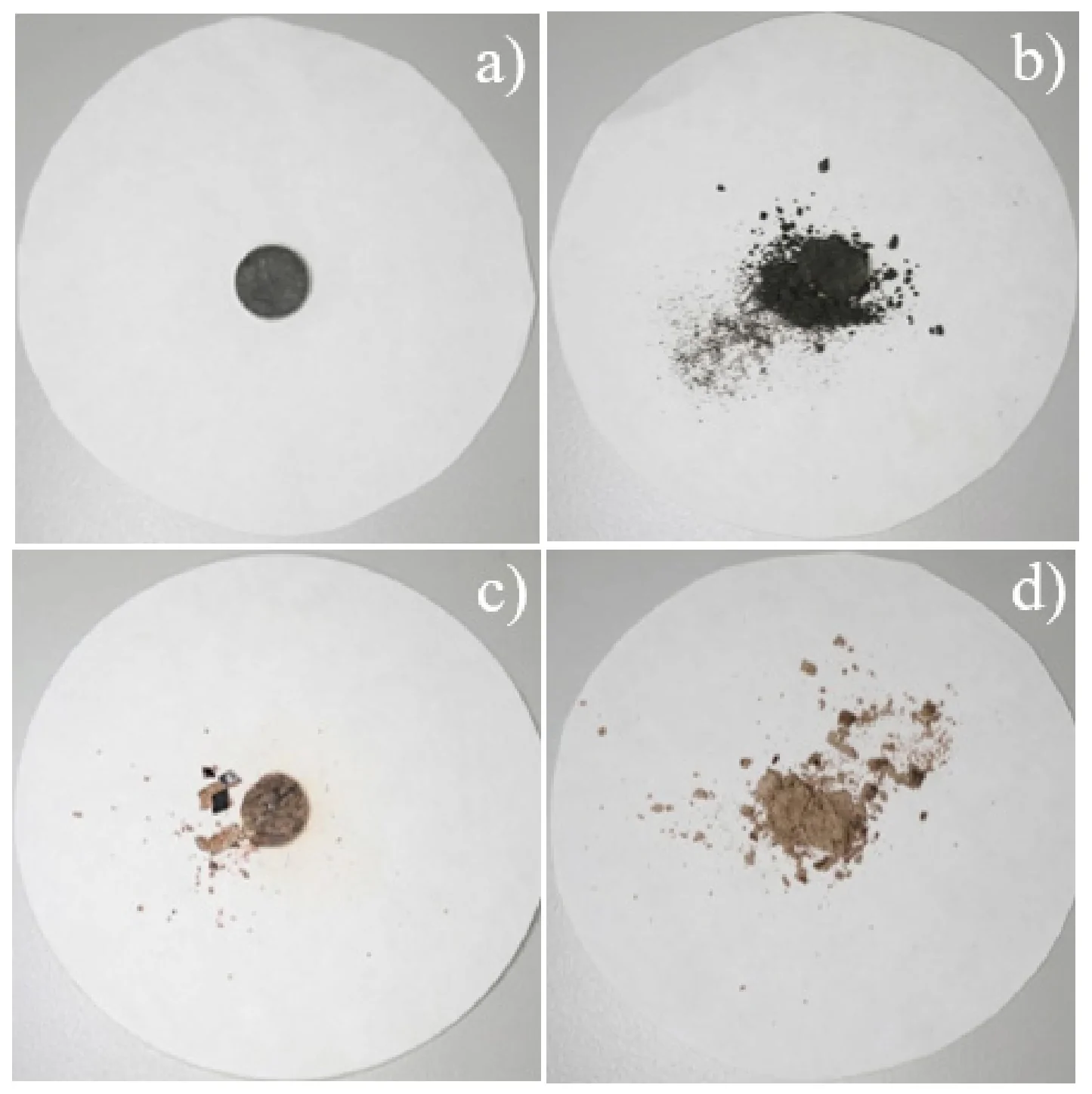
Appearance of the samples after exudation tests: (**a**) Premium, IPS at 0.3 MPa; (**b**) Absorbent, IPS at 0.3 MPa; (**c**) Premium, MP at 0.1 MPa; (**d**) Absorbent, MP at 0.1 MPa.

Before testing, the samples were ground to level the surface and ensure uniform load distribution during pressing. As a result, the sample mass before the start of the test (m_0_, Table 6) was lower than the mass recorded after completion of thermal treatment (Table 5), since part of the material was mechanically removed during surface grinding rather than due to hydrocarbon-phase loss during the exudation test itself. The exudation tests demonstrated a dependence of the mechanical stability of the samples on the thermal treatment condition and the material structure (Table 6). The appearance of the filter paper after the tests is shown in Figure 5.

Here, m_0_ is the mass of the prepared sample, while Δm_1_ and Δm_2_ are the mass losses at loads of 0.1 and 0.3 MPa, respectively. The test was performed on one sample for each sorbent and treatment condition. The sample was sequentially subjected to loads of 0.1 and 0.3 MPa with intermediate weighing. Exudation was assessed by the appearance of oil on the filter paper and changes in the sample mass during loading. The condition of the sample was recorded during the test. The tests were performed on one sample for each sorbent and treatment condition. After the test, the geometry and microhardness were not determined again due to partial or complete destruction of some samples.

The samples after pressure-assisted thermomechanical consolidation retained their integrity at a load of 0.1 MPa without any signs of mass loss. When the load was increased to 0.3 MPa, the Premium sample retained its integrity, whereas the Absorbent sample partially fractured, which may be associated with the presence of macrocracks identified by SEM. The samples after thermal treatment without applied pressure demonstrated lower resistance to mechanical loading. Fracture of the samples was observed already at a load of 0.1 MPa, indicating insufficient strength of the formed structure. Such behavior may be associated with a higher degree of densification of the material after pressure-assisted thermomechanical consolidation [29,43,47]. Two levels of fracture should be distinguished. For the Absorbent sample after pressure-assisted thermomechanical consolidation, partial fracture was observed at 0.3 MPa while the sample retained its overall shape, allowing the mass loss to be determined gravimetrically (Δm_2_, Table 6). After thermal treatment without applied pressure, complete structural failure occurred at 0.1 MPa, making accurate determination of the mass loss impossible. This type of catastrophic failure makes heat treatment without applied pressure unsuitable for sorbents with a high gypsum-phase content under actual conditions of storage and transportation of conditioned waste.

### 3.6. Microhardness Study

The degree of densification of the samples was evaluated based on the results of Vickers microhardness measurements (Table 7).

The wider spread of values for Absorbent (range of 32.20 HV, coefficient of variation of 20.5%) compared with Premium (range of 2.71 HV, coefficient of variation of 10.4%) is consistent with the presence of macrocracks and structural heterogeneity identified by SEM (Section 3.2), whereas the narrower spread for Premium reflects its more homogeneous morphology. Direct comparison of microhardness with apparent density (Table 5) does not reveal an unambiguous relationship, since the higher apparent density of the Absorbent sample is determined primarily by the higher true density of the initial raw material rather than by more effective mechanical densification of the structure.

Despite the higher microhardness values of the Absorbent sample, the exudation tests did not show a direct relationship between mechanical stability and material hardness. This indicates that mechanical stability is determined not only by the hardness value but also by the structural features of the material. The preservation of the shape and integrity of the Premium samples under mechanical loading is an important factor for reliable retention of the hydrocarbon phase within the matrix and ensuring the non-flowing state of the material [21,48,49].

The key marker explaining the differences in the behavior of the sorbents was the presence and relative predominance of the gypsum phase, identified by XRD [47]. The SEM and EDS data confirmed the physical manifestation of this difference, namely the formation of macrocracks and the distribution of elements characteristic of gypsum, while the gravimetric analysis and exudation tests quantitatively reflected its practical consequence in terms of differences in hydrocarbon-phase retention and mechanical stability. The specified composition of commercial sorbents does not always reflect their actual mineral composition, which emphasizes the need for XRD analysis before their application in technologies for the conditioning of oil-containing RAW. Based on this relationship, a practical criterion for the selection of aluminosilicate sorbents for the conditioning of oil-containing RAW can be formulated [50,51,52,53].

## 4. Conclusions

The present study established the effect of low-temperature thermomechanical consolidation on the formation of the material structure, hydrocarbon-phase retention, and mechanical stability of oil-saturated aluminosilicate sorbents. It was shown that the combined effect of temperature and uniaxial pressure provides the required formation of a consolidated structure at 150 °C for 20 s without preliminary removal of the oil phase or the introduction of additional binding components. Compared with thermal treatment without external pressure at the same temperature, this approach reduced the total mass losses from 0.74 to 0.35 g for Premium and from 0.75 to 0.47 g for Absorbent, while also resulting in a more pronounced increase in the calculated degree of densification.

The analysis performed using XRD, SEM, and EDS showed that the consolidation efficiency depends on the condition of the initial sorbent. For Premium, characterized by a predominance of the illite–montmorillonite component, the formation of a more homogeneous surface morphology and a reduction in the number of visible voids. In the case of Absorbent, for which a pronounced presence of the gypsum phase was established by XRD, thermomechanical treatment was accompanied by the formation of macrocracks. The obtained results indicate that, during the consolidation of oil-saturated aluminosilicate materials, not only the degree of densification but also the mineral composition of the initial sorbent is of critical importance.

The results of the mechanical tests additionally showed no direct correlation between microhardness and the macroscopic stability of the consolidated material. Despite the substantially higher microhardness of Absorbent, 44.28 ± 9.06 HV, compared with Premium, 8.37 ± 0.87 HV, the Premium sample retained its structural integrity at a load of up to 0.3 MPa, whereas Absorbent fractured at 0.3 MPa. For the samples after thermal treatment without applied pressure, fracture was observed already at 0.1 MPa. Thus, the mechanical stability of the investigated materials is determined by the combined contribution of the presence of the brittle gypsum phase, the nature of interparticle interaction, and the defects in the formed structure, whereas microhardness primarily characterizes the local resistance of the surface to deformation. This result is of fundamental importance for the selection of sorbents in technologies for conditioning oil-containing radioactive waste. Surface hardness provided by the presence of a brittle gypsum phase is not an indicator of material suitability, whereas crack resistance provided by the plasticity of the illite–montmorillonite matrix determines the ability of the material to maintain its integrity and prevent the release of a free hydrocarbon phase under mechanical loading during waste storage and transportation.

Taken together, the results demonstrate that low-temperature thermomechanical consolidation is a promising approach for forming a non-flowing and mechanically stable state of oil-saturated aluminosilicate sorbents. A fundamentally important result is the establishment of a relationship between the presence and relative predominance of the gypsum phase in the initial sorbent, the nature of structural densification, and the stability of hydrocarbon-phase retention. From a practical perspective, the results substantiate the need for preliminary mineralogical characterization of sorbents, including XRD analysis, prior to their use in technologies for the conditioning of oil-containing RAW. Further validation of the proposed approach using real oil-containing RAW, assessment of the radiation and thermal resistance of the consolidated forms under exposure to actual radionuclides, and assessment of the long-term stability of the consolidated forms, as well as testing of series of samples with a sufficient number of independent replicates and subsequent quantitative mechanical analysis after loading, should be considered necessary stages in the technological validation of the developed method.

## Figures and Tables

**Table 1:** Physicochemical characteristics of the studied sorbents.

Parameter	Sorbent Premium	Sorbent Absorbent
Main composition	Bentonite (90–100%)	Bentonite (90–100%)
Crystalline SiO_2_	0–10%	0–10%
Relative density	2.2	2.2
Bulk density	~0.504 g/cm^3^	~0.430–0.480 g/cm^3^
Specific volume	~1.98 cm^3^/g	~2.10–2.30 cm^3^/g

**Table 2:** Characteristics of the oil.

Property	Value
Hydrocarbon composition	Cycloparaffins ~70%, aromatic hydrocarbons 15–20%, paraffins 10–15%
Flash point (closed cup)	135–150 °C
Pour point	≤−45 °C
Acid number	<0.02 mg KOH/g
Impurities	Asphalt-resinous substances (up to 2%), S and N compounds (<1%)
Autoignition temperature	350–400 °C
Molecular weight	240–340 a.m.u.
Density (at 20 °C)	870–890 kg/m^3^
Kinematic viscosity (at 20 °C)	28.0–30.0 mm^2^/s
Kinematic viscosity (at 50 °C)	≤9.6 mm^2^/s

**Table 5:** Mass losses and calculated density and degree of densification of the consolidated samples.

Parameter	Premium (Under Pressure)	Premium (Without Pressure)	Absorbent (Under Pressure)	Absorbent (Without Pressure)
m_0_	2.00	2.00	2.00	2.00
m_1_ (after pressing), g	1.89	1.30	1.96	1.38
Δm_1_ (loss during pressing), g	0.11	0.70	0.04	0.62
m_2_ (after thermal treatment), g	1.65	1.26	1.53	1.25
Δm_2_ (loss during thermal treatment), g	0.24	0.04	0.43	0.13
Δm (total loss), g	0.35	0.74	0.47	0.75
Apparent density of the consolidated sample, g/cm	1.65	1.41	2.09	1.71
Degree of densification, %	31.50	12.70	30.70	6.4

**Table 6:** Results of exudation tests of samples after pressure-assisted thermomechanical consolidation and thermal treatment without applied pressure.

Sample	m_0_	m_1_	Δm_1_ = m_0_ − m_1_	m_2_	Δm_2_ = m_0_ − m_2_	Note
Premium (ISKRA)	1.6022	1.6022	0	1.5981	0.0041	Stable at 0.3 MPa
Absorbent (ISKRA)	1.4237	1.4237	0	1.4225	0.0012	Fractured at 0.3 MPa
Premium (muffle furnace)	1.0698	1.0670	0.0028	–	-	Fractured at 0.1 MPa
Absorbent (muffle furnace)	0.9338	Fractured	–	–	–	Fractured at 0.1 MPa. Accurate mass measurement was impossible.

**Table 7:** Results of hardness measurements of the samples after pressure-assisted thermomechanical consolidation.

Sorbent	Test Load, N	Dwell Time, s	Mean Vickers Hardness, HV ± Standard Deviation	Min., HV	Max., HV	CV, %
Premium (ISKRA)	2.942 (20× objective)	15	8.37 ± 0.87	7.69	10.40	10.4
Absorbent (ISKRA)	9.807 (20× objective)	13	44.28 ± 9.06	33.60	65.80	20.5

## Data Availability

The original contributions presented in this study are included in the article. Further inquiries can be directed to the corresponding authors.
